# Comparative hydrodynamic and nanoscale imaging study on the interactions of teicoplanin-A2 and bovine submaxillary mucin as a model ocular mucin

**DOI:** 10.1038/s41598-023-38036-6

**Published:** 2023-07-13

**Authors:** Taewoo Chun, Jacob Pattem, Richard B. Gillis, Vlad T. Dinu, Gleb E. Yakubov, Anthony P. Corfield, Stephen E. Harding

**Affiliations:** 1 National Centre for Macromolecular Hydrodynamics, University of Nottingham, Sutton Bonington, LE12 5RD UK; 2 Soft Matter Biomaterials and Biointerfaces, School of Biosciences, University of Nottingham, Sutton Bonington, LE12 5RD UK; 3 College of Business, Technology and Engineering, Food and Nutrition Group, Sheffield Hallam University, Arundel Gate, Sheffield, S1 1WB UK

**Keywords:** Biochemistry, Biophysics, Microbiology, Diseases

## Abstract

Glycopeptide antibiotics are regularly used in ophthalmology to treat infections of Gram-positive bacteria. Aggregative interactions of antibiotics with mucins however can lead to long exposure and increases the risk of resistant species. This study focuses on the evaluation of potential interactions of the last line of defence glycopeptide antibiotic teicoplanin with an ocular mucin model using precision matrix free hydrodynamic and microscopic techniques: sedimentation velocity in the analytical ultracentrifuge (SV-AUC), dynamic light scattering (DLS) and atomic force microscopy (AFM). For the mixtures of teicoplanin at higher doses (1.25 mg/mL and 12.5 mg/mL), it was shown to interact and aggregate with bovine submaxillary mucin (BSM) in the distributions of both sedimentation coefficients by SV-AUC and hydrodynamic radii by DLS. The presence of aggregates was confirmed by AFM for higher concentrations. We suggest that teicoplanin eye drop formulations should be delivered at concentrations of < 1.25 mg/mL to avoid potentially harmful aggregations.

## Introduction

**Figure 1:**
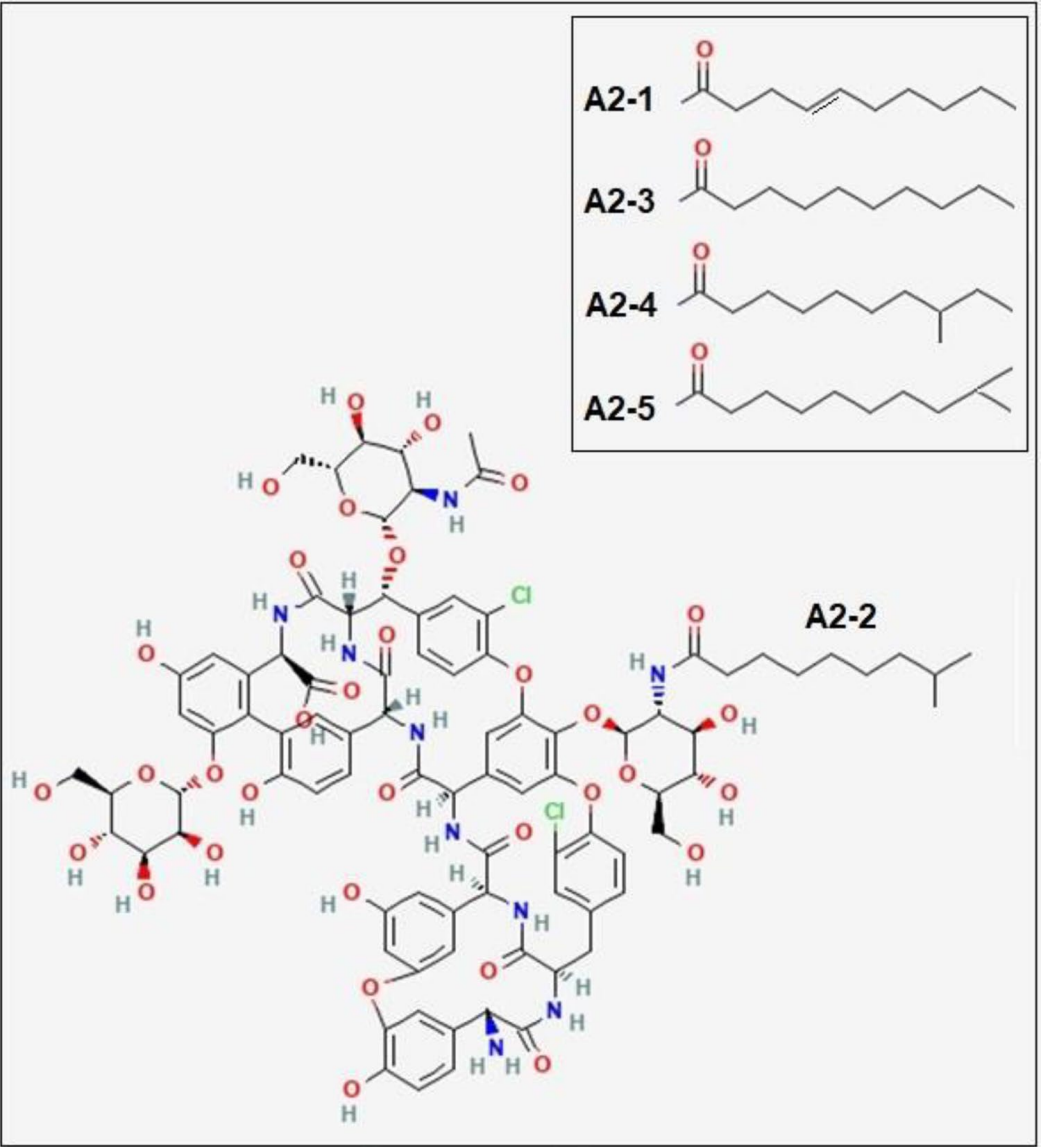
Structure of teicoplanin (adapted from the National Institute for Health/ National Centre of Biotechnology Information^10^ based on an original structure given by F. Parenti^11^) and teicoplanin lipoform A2-2 (*M*_1_ = 1879.7 g/mol) with the other major types of A2 subtypes with different acyl chains are shown in the inset.

Teicoplanin is a member of the glycopeptide antibiotics family, such as vancomycin, as the ‘last resort of defence’ drug to treat severe infections of Gram-positive bacteria including methicillin-resistant *Staphylococcus aureus* (MRSA) and enterococci^1^. Its chemical structure was first determined in 1984^2,3^. Figure 1 shows that the core aglycone structure of teicoplanin is a linear heptapeptide, combined with three monosaccharide residues: α-D-mannose, *N*-acetyl-β-D-glucosamine, and one of five subtypes of *N*-acyl-β-D-glucosamines depending on teicoplanin A2-1 through A2-5^4^. The mixture of five subtypes of teicoplanin is produced by *Actinoplanes teicomyceticus*^5^ and is generally administered as a single product in clinical practice^6^. The prominent difference with vancomycin is the presence of a long fatty acid chain attached to the (*N*-acyl-) β-D-glucosamine residue, compared with vancomycin bearing a non-acylated disaccharide residue^7^. Both glycopeptide antibiotics show antibacterial activity in the same manner. Namely, they bind to bacterial membranous protein (Lipid II) preventing this peptidoglycan precursor from insertion into the bacterial cell wall^8^. However, to increase their affinity for the peptidyl D-Ala-D-Ala motif of Lipid II, vancomycin cooperatively dimerises in a back-to-back manner, while teicoplanin shows a much greater tendency to self-associate: in the concentrations 0–10 mg/mL teicoplanin A2 was found to self-associate plateauing > 1 mg/mL to give a molar mass of (35,400 ± 1000) g/mol corresponding to ~ (19 ± 1) mers, with a sedimentation coefficient^9^ s_20, w_ =  ~ 4.65 S.

Bacterial infection of the eyes ranges from relatively easily treated conditions, such as conjunctivitis and blepharitis^12^, to more serious ones including keratitis^13^, and notoriously endophthalmitis^14^. The significance of MRSA strains for ocular infections is on the increase. For example, Harford et al.^15^ reported that in the U.K. the percentage of MRSA-positive cases from eye swabs was 2% on average in 2013–2019. Those cases are rising in the U.S.^16^ and South India^17^.

Therefore, the eradication of MRSA strains on ocular surfaces is routinely performed for proven positive patients in pre-operative screening. Those patients receive chloramphenicol drops before eye surgery and intracameral vancomycin intraoperatively^15^. Vancomycin eye drops (50 mg/mL) are also used for the treatment of MRSA-positive keratitis^18^. On the other hand, teicoplanin drops are reported to have no or little corneal penetration in rabbits^19^ and in patients at 10 mg/mL^20^. For orbital cellulitis, intravenous teicoplanin can be applied to patients with penicillin allergy, along with oral ciprofloxacin and metronidazole^21^.

However, the emergence of further resistant species against these glycopeptide antibiotics becomes a burning issue for antibiotic selection in ocular infections. Vancomycin- and teicoplanin-resistant enterococcal species were reported in the U.S.^22^ and Europe^23,24^ before the 1990s. Resistance gene clusters are transferred between bacterial cells through plasmids^6^. Glycan antibiotics attach to the D-Ala-D-Ala motif of Lipid II through five hydrogen bonds, though these resistance operons modify that targeted motif into D-Ala-D-Lac for *vanA*, *vanB*, *vanD*, *vanF*, and *vanM*, and D-Ala-D-Ser for *vanC*, *vanE*, *vanG*, *vanL*, and *vanN*^25^. This modification reduces the number of hydrogen bonds and Lipid II affinity. Overuse and misuse of antibiotics are considered major causes of antimicrobial resistance^26^. This is due to excessive exposure of bacterial populations to antibiotics. These human activities lead to selection pressure, increasing resistance genes in microbes^27^.

Ocular mucins may also contribute to antimicrobial resistance (AMR). There is no doubt that the mucin layer of tear films protects against bacterial adherence^14^. It is reported that ocular mucus, as well as bovine submaxillary mucin (BSM), inhibited the adherence of *Pseudomonas aeruginosa* on the rabbit corneal epithelium^28^. However, it is also reported that antibiotics bind to mucins of intestinal^29^ and respiratory^30^ tracts, showing a substantial reduction of their microbial activity^31^. Samad et al.^32^ suggested that mucin glycoproteins (MUC5AC, MUC2, and MUC5B) interacted with two antibiotics against gram-positive *P. aeruginosa* (polymyxin and fluoroquinolone), increasing its growth after exposure to antibiotics. Dinu et al.^33^ reported that gastrointestinal mucins and BSM induced aggregation with vancomycin. Therefore, mucin components of tear films might also exacerbate AMR through their interactions with antibiotics.

In this study, we use commercially available BSM as a popularly used model ocular mucin to explore potential interaction and aggregation behaviour with teicoplanin. BSM – which in common with ocular mucin has a lower degree of glycosylation compared with respiratory, reproductive and alimentary tract mucins—has been widely used as a tear film model for the evaluation of contact lenses^34–36^ and for the interactions with polysaccharides^37,38^ and proteins^39^. Setälä et al.^39^ reported the validity of BSM as an ocular mucin model for in vitro interaction experiments, pointing out that both ocular mucins and BSM interacted with phospholipid transfer protein. In contrast, it should be noted that BSM may not necessarily behave like ocular mucins, such as MUC5AC, the most abundant and gel-forming mucin since commercially available BSM is not a gel-forming mucin^40^. Furthermore, Rivera and Tessarollo^41^ warned against dependence on a single animal model to extrapolate its findings to human pathophysiology, such as human carcinogenesis^42^ and inflammatory diseases^43,44^. However, it is worth conducting in vitro interaction experiments with BSM as a substitute for whole mucin components of tear films before sufficiently collecting and purifying human ocular mucins.

This interaction study is based on a relatively novel combination of hydrodynamic and microscopic methods. Sedimentation velocity in the analytical ultracentrifuge (SV-AUC) is the gold standard method used to evaluate the integrity of various macromolecules, such as glycoproteins^45^. SV-AUC provides sedimentation coefficients and distributions relating to molecular sizes^46^. Dynamic light scattering (DLS) also provides macromolecular sizes complementary to SV-AUC data^47^. The hydrodynamic results can be confirmed by visualising macromolecular aggregates from samples. Atomic force microscopy (AFM) can detect morphological dynamic changes on a nanometre scale^48^. AFM is a powerful tool for visualising nanostructure and has been used in studies with ocular mucin aggregation^49^ and gastric mucin-chitosan interactions^50^.

The combination of hydrodynamic and microscopic methods was first used by Dinu et al.^33^ to assess the aggregation of vancomycin with mucins, such as BSM. Many studies have been designed to focus on how glycan antibiotics bind to Lipid II^51^ and the genomic profiles of resistant microbes^32^, though the study on environmental factors including mucin-antibiotic binding is of great importance. The hydrodynamic and microscopic methods will also evaluate teicoplanin regarding the degree of interactions with BSM as an outer mucus model.

## Methods

### Teicoplanin and Bovine submaxillary mucin, BSM

Teicoplanin A2 powder (the mixture of teicoplanin A2-1 with monomer molar mass *M*_1_ = 1877.6 g/mol, teicoplanin A2-2 and A2-3 with *M*_1_ = 1879.7 g/mol, and teicoplanin A2-4 and A2-5 with *M*_1_ = 1893.7 g/mol) was obtained from Sigma-Aldrich, the United Kingdom. A refractive increment *dn/dc* of 0.188 mL/g was used^52^. The stock solution concentration was then measured with a differential refractometer (Atago DD7, Tokyo, Japan). The final concentrations (0.125 mg/mL, 1.25 mg/mL, and 12.5 mg/mL) of teicoplanin A2 were prepared in a phosphate-chloride buffered saline solution (PBS or “Paley buffer”) at pH ~ 6.8, ionic strength of I = 0.1 mol/L^53^.

BSM (Sigma-Aldrich, U.K., catalogue no. M3895, type I-S) was purchased and dissolved in the previously described PBS buffer. A refractive increment *dn/dc* of 0.181 mL/g for BSM^54^ was used. The stock solution concentration was then determined with the differential refractometer (Atago DD7, Tokyo, Japan).

#### Sedimentation velocity in the analytical ultracentrifuge (SV-AUC)

SV-AUC experiments were conducted at 20.0 °C with the Optimal XL-I analytical ultracentrifuge (Beckman, Palo Alto, U.S.A.) coupled to Rayleigh interference optics. Reference solvent (PBS) of 420 μL and sample solutions (teicoplanin A2, BSM, and mixtures of teicoplanin A2 and BSM) of 400 μL were injected into the channels of 12 mm double-sectored cells with sapphire windows and rotated at 47,500 rpm for a run time of ~ 24 h until the specimen completely sedimented. The data was then obtained by the interference system to monitor changes in the concentration of samples in fringe units over radial displacement. This involves the acquisition of multiple (> 50) radial scans as a function of time from which accurate sedimentation coefficient distributions are obtained with the SEDFIT algorithm^55^.

This algorithm produces by the least squares ls-g*(*s*) method the sedimentation coefficient distribution, *g(s)* versus *s*_T,b_, where *s* is the sedimentation coefficient at temperature *T* and in buffer *b*. The value of *s*_T,b_, given by the unit of Svedberg (S) = 10^–13^ s, was normalised to standard conditions with viscosity and density of water solvent at 20.0 °C, ${s}_{20,w}$, using the equation ^56^:

1 $${s}_{20,w}=\frac{1 - \overline{\upsilon }\cdot {\rho }_{20,w}}{1 - \overline{\upsilon }\cdot {\rho }_{T,b}}\cdot \frac{{\eta }_{T,b}}{{\eta }_{20,w}}\cdot {s}_{T,b}$$

where ${\rho }_{T,b}$ and ${\eta }_{T,b}$ are the density and the viscosity of buffer *b* at temperature *T*, respectively. The following sets of samples were used: 1 mg/mL BSM control (high enough to give a good signal but still clear of molecular overlap), teicoplanin A2 control (0.125 mg/mL, 1.25 mg/mL, and 12.5 mg/mL), and the mixtures (1 mg/mL BSM + 0.125 mg/mL teicoplanin A2, 1 mg/mL BSM + 1.25 mg/mL teicoplanin A2, and 1 mg/mL BSM + 12.5 mg/mL teicoplanin A2).

### Dynamic light scattering (DLS)

DLS experiments were performed using a fixed scattering angle Zetasizer Nano-S system (Malvern Panalytical Ltd., Malvern, U.K.) coupled to a 4 mV He–Ne laser at 632.8 nm^57,58^. Sample measurements were performed in a 1 cm square cuvette at 20.0 °C using a scattering angle of 173°. The data were obtained and analysed with the “Zetasizer Software (Version 7.1)” (Malvern Panalytical Ltd, Malvern, U.K.). For each sample analysed multiple autocorrelation profiles were obtained for 10 scans per 3 replicates averaged over 10 min. The CONTIN programme^59^ gives volume distributions of translational diffusion coefficients, *D*_z_. The z-average hydrodynamic radii, *r*_z_, (nm) were then determined from *D*_z_ using the Stokes–Einstein equation^57^:

2 $${r}_{z}=\frac{{k}_{B}T}{6\pi \eta {D}_{z}}$$

where k_B_ is the Boltzmann constant. In these experiments, non-ideality effects were assumed to be insignificant because the sample solutions are sufficiently dilute and sample sizes are small. Therefore, an extrapolation to zero concentration is unnecessary. Additionally, for translational diffusion, non-ideality is related to the two major factors in the hydrodynamic and thermodynamic terms, though these factors can compensate for and thus cancel each other^60,61^. Moreover, the teicoplanin samples–whether monomeric or multimeric forms in solution–were assumed not to be asymmetric. Therefore, the measured values of *D*_z_ were independent of the angle and extrapolation to zero angle to removed rotational diffusion/ anisotropic effects was unnecessary^57^.

### Atomic force microscopy (AFM) analysis

A Dimension ICON (Bruker Nano, Santa Barbara, CA, USA) using dedicated software (Nanoscope 9.4) was used to image 3 independent 500 nm × 500 nm areas of teicoplanin (1.25 mg/mL), BSM (1 mg/mL), and teicoplanin-BSM at increasing teicoplanin concentrations of 0.125 mg/mL, 1.25 mg/mL, and 12.5 mg/mL. All specimens were prepared by depositing 10 µL on cleaved mica and left to air dry at room temperature for 24 h before imaging. RTESPA-150 (Bruker Nano, Santa Barbara, CA) cantilevers were used across all samples, imaging in Tapping mode™ operating at a resonance frequency of 150 kHz in air at room temperature. Particle size analysis was performed using Nanoscope Analysis Version 1.9 monitoring height (nm) and diameter (nm) across all analysed samples. Statistical analysis between teicoplanin and BSM-teicoplanin at increasing teicoplanin concentrations was performed using a series of Kruskal–Wallis ANOVAs.

## Results

### Analytical Ultracentrifugation (AUC) of teicoplanin-BSM solutions

**Figure 2:**
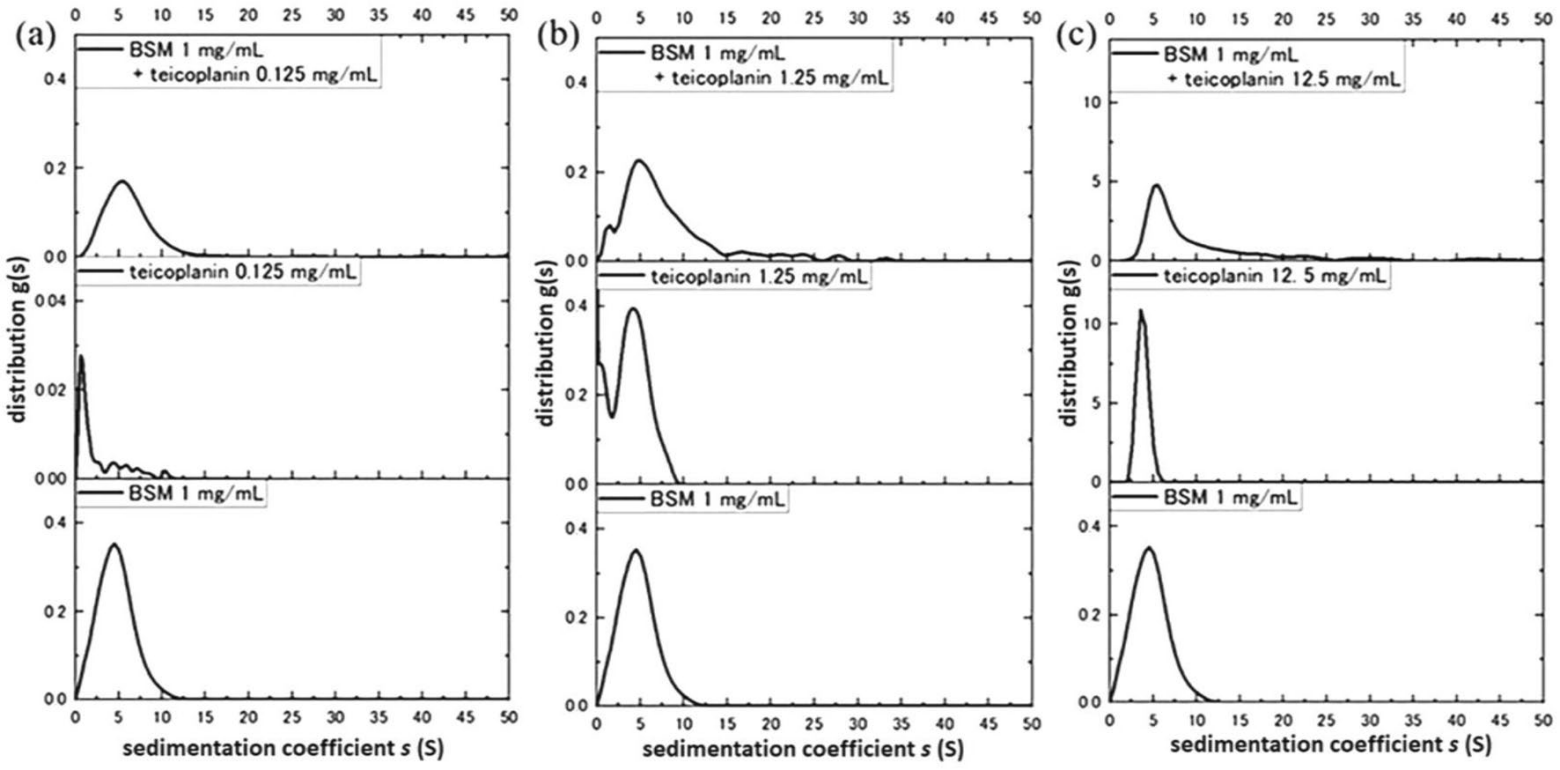
Sedimentation coefficient distributions of mixtures of 1 mg/mL BSM with (**a**) 0.125 mg/mL teicoplanin (**b**) 1.25 mg/mL teicoplanin, and (**c**) 12.5 mg/mL teicoplanin (**c**). Solutions in phosphate-chloride buffer pH 6.8, I = 0.10. Rotor speed 47,500 rpm, at 20.0 °C.

Figure 2 shows the sedimentation coefficient distributions for the interactions of teicoplanin with BSM 1 mg/mL. The values of ${s}_{20,w}$ for the BSM control was ~4.6S, and teicoplanin controls of 0.125 mg/mL (Fig. 2a), 1.25 mg/mL (Fig 2b), and 12.5 mg/mL (Fig. 2c) are ~0.9S (0.125 mg/mL teicoplanin), ~4.5S (1.25 mg/mL teicoplanin) and ~3.3S (12.5 mg/mL teicoplanin). For the mixture of BSM and 0.125 mg/mL teicoplanin (Fig. 2a), there was no clear interaction between each component (${s}_{20,w}$ of the mixture was ~ 5.4S).

On the other hand, there were clearer shifts for BSM with 1.25 mg/mL teicoplanin (${s}_{20,w}$ of the mixture ~5.9S), and for BSM with 12.5 mg/mL teicoplanin (${s}_{20,w}$ of the mixture was ~6.3S). Furthermore, some aggregates emerged over far higher values (20–40S) under the presence of the mucin. For Fig. 2b, there was a loss of the teicoplanin peak at ~0.9S (teicoplanin unimer) in the mixture, suggesting that all teicoplanin unimers were exhausted by complexation with BSM.

### Dynamic light scattering (DLS)

**Figure 3:**
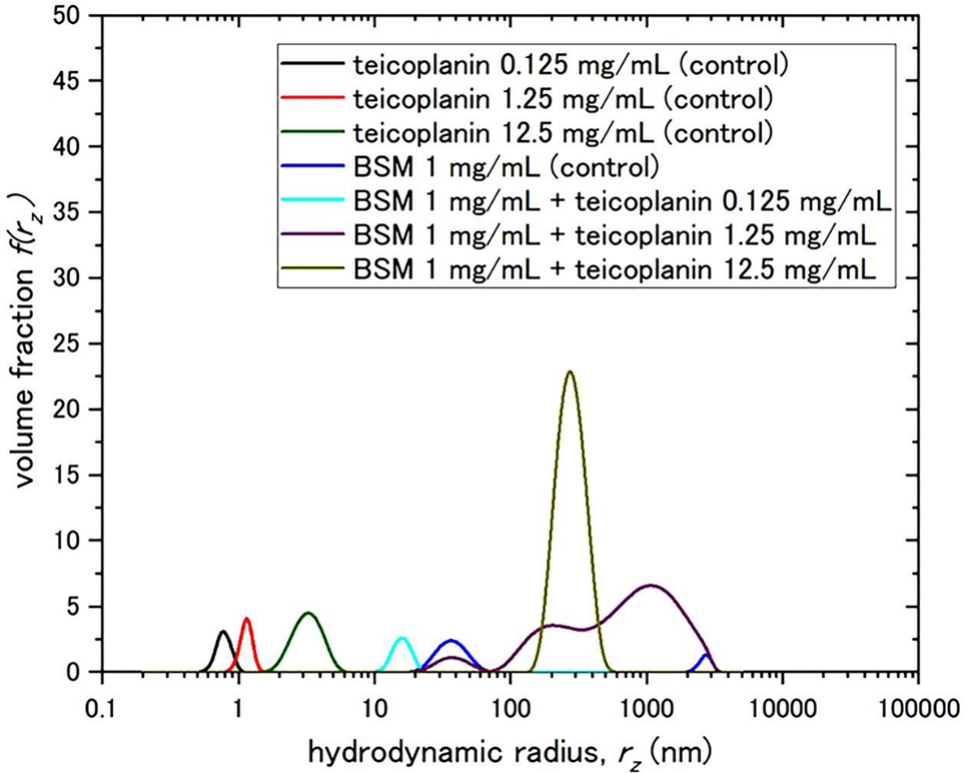
The distributions of z-average apparent hydrodynamic radii, *r*_z_ from DLS measurements on 1 mg/mL BSM with teicoplanin 0.125 mg/mL, 1.25 mg/mL, and 12.5 mg/mL. The different colours denote each sample: teicoplanin controls (0.125 mg/mL for the black line, 1.25 mg/mL for the red line, and 12.5 mg/mL for the green line), BSM control (violet line), the mixture of BSM and 0.125 mg/mL (sky blue line), the mixture of BSM and 1.25 mg/mL (purple line), and the mixture of BSM and 12.5 mg/mL (dark yellow line).

Further evidence of BSM-teicoplanin aggregation is shown in Fig. 3 providing the distributions of apparent hydrodynamic radii (after transformation from the apparent translational diffusion coefficients through the Stokes–Einstein equation, Eq. (2)). Distributions were obtained from software based on the CONTIN algorithm of Provencher^59^.

There was a partial loss of mucin components (observed at ~ 30 nm and 3000 nm in the control) in the mixture of BSM and 1.25 mg/mL teicoplanin. There was also a complete loss of these two components in the mixture of BSM with 12.5 mg/mL teicoplanin, and new aggregates emerge at 300 nm. These results from DLS experiments appear to reinforce the observations of SV-AUC in terms of the interaction/ aggregation behaviour of the mixtures.

The apparently higher proportion of aggregate appearing in DLS is caused by the disproportionate higher scattering of larger particles, compared to smaller ones: this does not affect the sedimentation velocity in the same way, where the optical detection system is based on interference from refractive index differences between solvent and solution, and it gives a much more effective separation of particles of different size.

### AFM Imaging of teicoplanin-BSM aggregates

**Figure 4:**
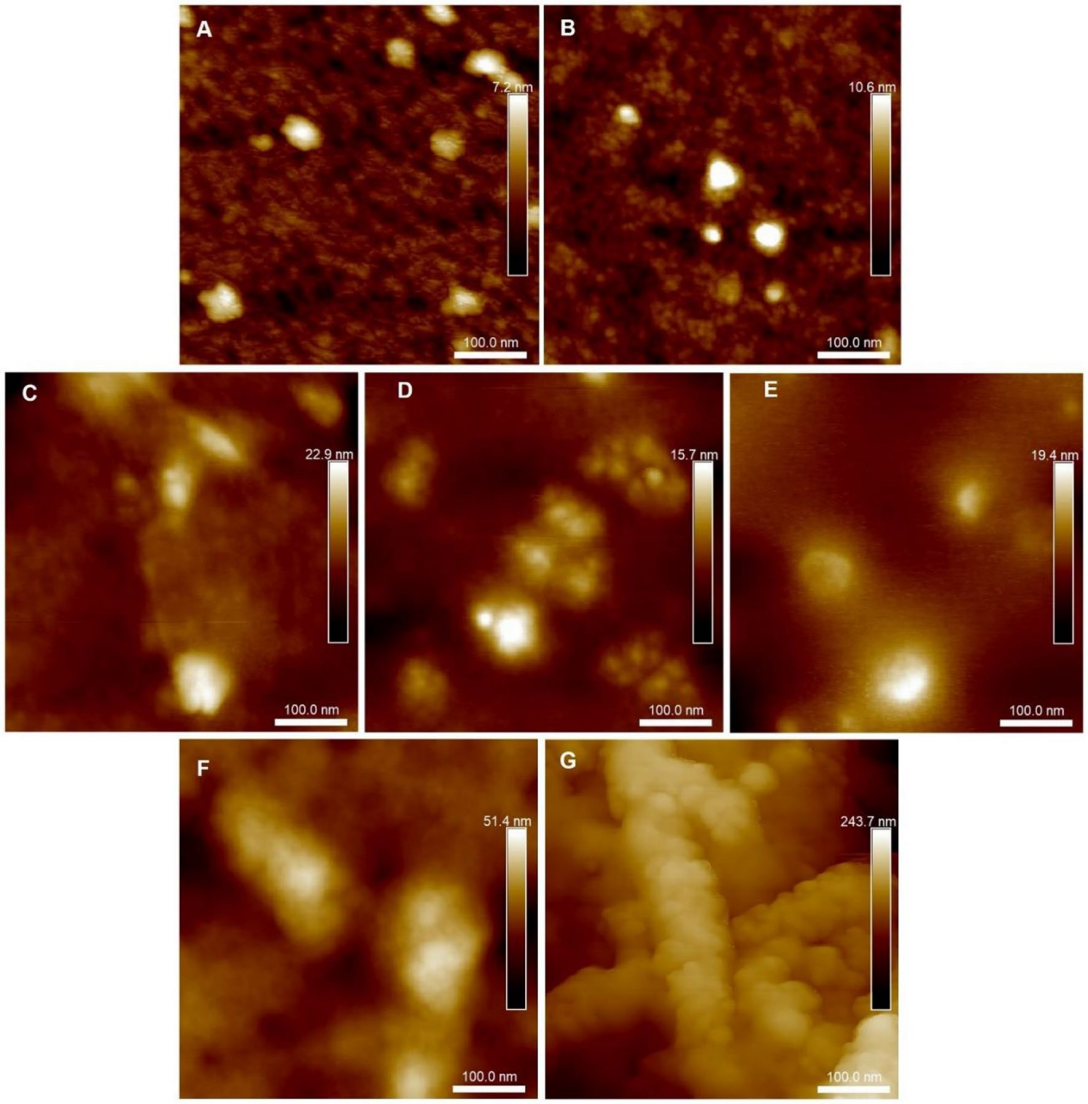
Atomic Force Microscopy (AFM) images (500 nm x 500 nm) of (**A**) teicoplanin 0.125 mg/mL, (**B**) teicoplanin 1.25 mg/ml (**C**) teicoplanin 12.5 mg/mL, (**D**) BSM 1 mg/mL, (**E**) teicoplanin-BSM 0.125 mg/mL, (**F**) teicoplanin-BSM 1.25 mg/mL and (**G**) teicoplanin-BSM 12.5 mg/mL.

Further evidence comes from atomic force microscopy. Figure 4A–C (and Supplementary Information) shows example 500 nm × 500 nm AFM images of teicoplanin A2 samples at increasing concentrations of 0.125 mg/mL, 1.25 mg/mL, and 12.5 mg/mL, with BSM 1 mg/ml shown in Fig. 4D. Figure 4E–G shows BSM and teicoplanin mixtures at increasing concentrations of teicoplanin at 0.125 mg/mL, 1.25 mg/mL and 12.5 mg/mL respectively. It is clear from the AFM imaging an observable increase in particle size is obtained when increasing the concentration of Teicoplanin and Teicoplanin in the BSM mixtures shown in Fig. 4A–G: Table 1 shows the average particle height (nm) and diameter (nm) for each samples, from 3 independent areas across the sample cohort.

**Table 1:** Average particle height (nm) and diameter (nm) for all analysed samples and concentrations. *p < 0.05 for comparison between TP (teicoplanin) 0.125 mg/mL, TP 1.25 mg/mL. TP 12.5 mg/mL, BSM-TP 0.125 mg/mL and BSM-TP 1.25 mg/mL.

Sample	Average height (nm)	Average diameter (nm)
TP control (0.125 mg/mL)	3.0 ± 0.6	26.2 ± 6.3
TP control (1.25 mg/mL)	8.3 ± 1.6*	29.5 ± 5.9
TP control (12.5 mg/mL)	16.2 ± 4.5*	80.9 ± 24.6*
BSM Control (1 mg/mL)	7.2 ± 5.2	83.8 ± 46.7
BSM-TP (0.125 mg/mL)	6.6 ± 1.3	44.1 ± 3.3
BSM-TP (1.25 mg/mL)	19.5 ± 1.9*	150.5 ± 30.3*
BSM-TP (12.5 mg/mL)	–	–

Teicoplanin alone at increasing concentrations of 0.125 mg/mL, 1.25 mg/mL and 12.5 mg/mL exhibited significant increases in particle height from (3.0 ± 0.6) nm to (8.3 ± 1.6) nm (*p* < 0.05) and (16.2 ± 4.5) nm (*p* < 0.05) respectively. This was also evident in particle diameter analysis although the only significant increase in diameter for teicoplanin controls was increasing the teicoplanin concentration from 1.25 mg/mL to 12.5 mg/mL (*p* < 0.05) as shown in Table 1. BSM-teicoplanin mixtures at increasing teicoplanin concentrations of 0.125 mg/mL and 1.25 mg/mL show significant increasing particle heights of (6.6 ± 1.3) nm and (19.5 ± 1.9) nm (*p* < 0.05) shown Table 1. This was also evident in particle diameter when increasing the concentration of teicoplanin in the BSM-teicoplanin mixtures from 0.125 mg/mL to 1.25 mg/mL, exhibiting (44.1 ± 3.3) nm and (150.5 ± 30.3) nm respectively (*p* < 0.05). At the greatest teicoplanin-BSM concentration, particle sizes could not be calculated as they exceed the size of the analysed image area (more than 500 nm). From the AFM imaging shown in Fig. 4 and particle size analysis in Table 1, it is evident aggregation is occurring with increasing teicoplanin concentration both alone and in the presence of BSM, consequently increasing particle size properties.

## Discussion

Teicoplanin A2 demonstrates clear interactions with BSM at concentrations > 1.25 mg/mL, based on independent orthogonal hydrodynamic and microscopic assessments. Additionally, there are some teicoplanin-BSM aggregates at > 1.25 mg/mL, preventing teicoplanin unimer from maintaining its antibiotic activity in a mucin-rich environment. Interestingly, unlike vancomycin which has a net positive charge under physiological conditions, both teicoplanin A2 and BSM molecules are negatively charged^54,62,63^. The hydrophobic force might be employed rather than the electrostatic forces with its acyl tail in terms of complexation. The partial interactions and aggregations of teicoplanin with BSM have three consequences, as follows–(1) since teicoplanin A2 uses its hydrophobic tail to allocate itself near the Lipid II precursor as a monomer^64^, the multimeric formation with mucins inevitably reduces its antimicrobial activity. (2) Also, since aggregates are more likely to be removed and transferred into nasolacrimal ducts by blinking^65^, total concentrations of teicoplanin decrease on ocular surfaces and as a result more frequent application of eye drops. (3) Most importantly, there is a good possibility that aggregations of teicoplanin with mucins result in longer exposure to antibiotics, leading to more selection pressure towards antimicrobial resistance between bacterial populations. Therefore, teicoplanin eye drops may be preferred to be at least < 1.25 mg/mL or more preferably, around 0.125 mg/mL, where there were no clear interactions and aggregates with BSM.

## Concluding remarks

The hydrodynamic data together with a high-resolution microscopic method–AFM, provide clear evidence of interactions between teicoplanin A2 with BSM resulting in the formation of undesirable aggregates, with a concentration-dependent growth in the size of these aggregates. We conclude that the appropriate concentrations of teicoplanin A2 eye drops based on this evidence using BSM as an ocular mucin model would be at least less than 1.25 mg/mL to avoid aggregational behaviours with mucins. The combination of the three independently orthogonal techniques–analytical ultracentrifugation (AUC), dynamic light scattering (DLS) and atomic force microscopy (AFM) has thus proven useful for understanding our model mucin system. AUC and DLS possess an advantage over many other methods for studying interactions of being matrix free and being pure solution techniques—i.e. not requiring separation columns or membranes or interaction with quartz interfaces or tethering to surfaces. AUC possesses the additional advantage of having an inherent separation and analysis facility. AFM permits the visualization of complexes, within the limitation of not being in a solution environment (even if a layer of liquid is placed on top). The three are very much complementary, hence the selection. Nonetheless the use of additional techniques in future work, such as surface plasmon resonance and quartz crystal microbalance measurements would help to elucidate the mechanism of the interaction (e.g. electrostatic interactions, etc.), quantify its kinetics and thus help to further corroborate our hypotheses.

Our experiments have been performed at 20.0 °C: further work at physiological temperatures may prove useful although the ocular surface is likely to be considerably less than 37 °C. More seriously, the validity of BSM as the ocular mucin model–although a good indicative first step–remains open to question for the study of antibiotics in ophthalmology. Many studies have used commercially available BSM as the ocular mucin model^34–36,39^, though there are some differences with ocular mucins, especially gel-forming mucins including MUC5AC. On the other hand, it is also reported that the mucus layer on the ocular surface is not the same as the truly gel-forming layer presented in respiratory and intestinal tracts, due to its relatively thin (1 μm) mucus layer^66^. Whatever the ocular mucus layer would be, it should be noted that differences with human ocular mucins also need to be explored in terms of the interaction study with teicoplanin. Ocular mucins have been shown to have virtually no surface activity^67^ and this may lead to reduced aggregation. This current study should therefore be regarded as providing the groundwork for further comparative studies—when evaluated in sufficient quantity—with ocular mucins.

## Supplementary Information

Supplementary Information 1.

Supplementary Information 2.

## Data Availability

The datasets used and analysed in the current study are supplied in the Supplementary Data and Supplementary Information files.
