# Supplementary material for: Comparative hydrodynamic and nanoscale imaging study on the interactions of teicoplanin-A2 and bovine submaxillary mucin as a model ocular mucin
Source: Sci Rep. 2023 Jul 13;13:11367. doi: 10.1038/s41598-023-38036-6 (PMC10344913; doi:10.1038/s41598-023-38036-6)

Comparative hydrodynamic and nanoscale imaging study on the interactions of teicoplanin-A2 and bovine submaxillary mucin as a model ocular mucin
(Chun et al)

Supplementary Information for Figure 4: AFM imaging for teicoplanin-BSM mixtures, with controls

Teicoplanin control 0.125 mg/ml – H_2O

Area 1

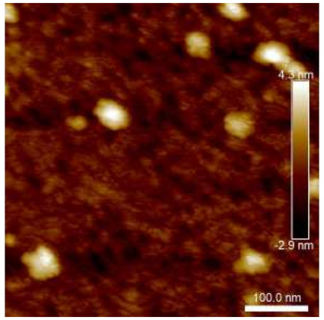

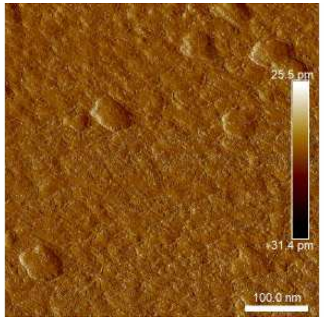

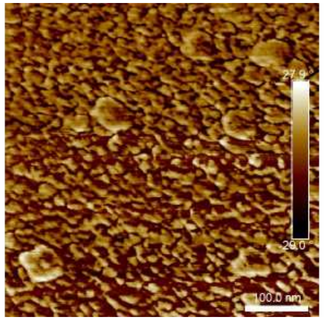

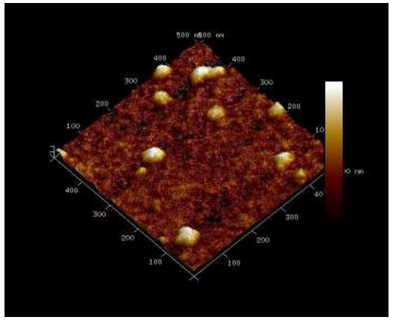

Area 2

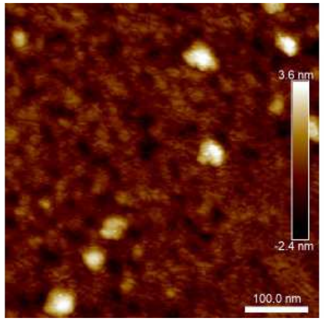

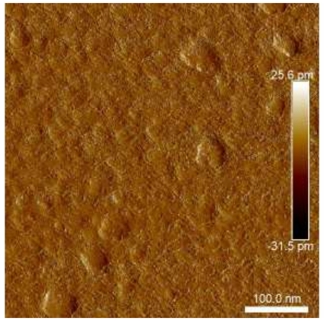

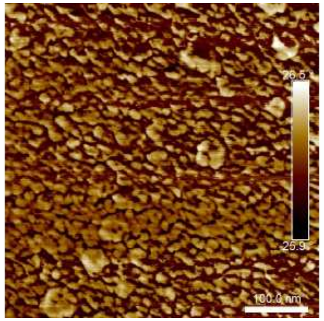

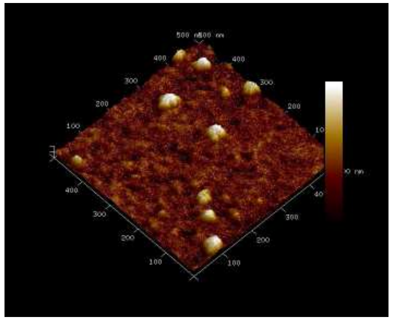

Area 3

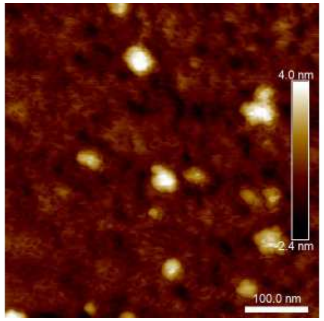

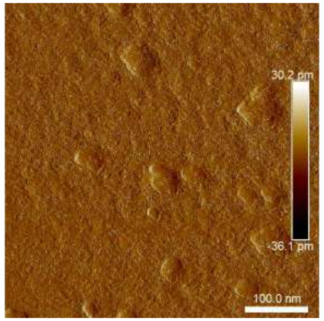

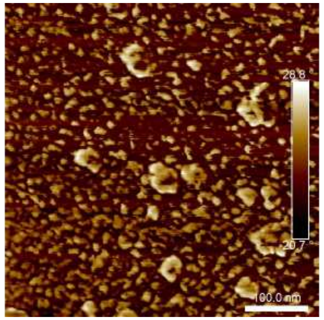

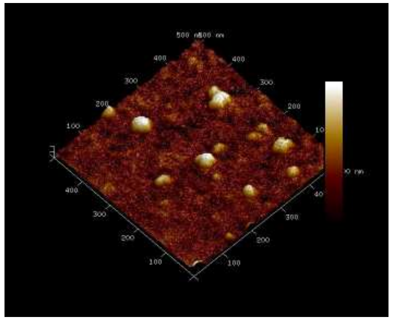

Teicoplanin Control 1.25 mg/ml – H_2O

Area 1

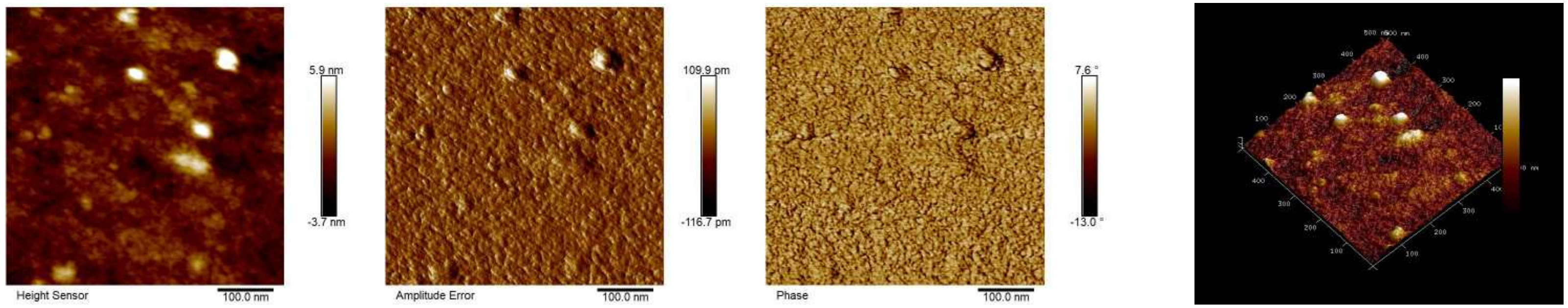

Area 2

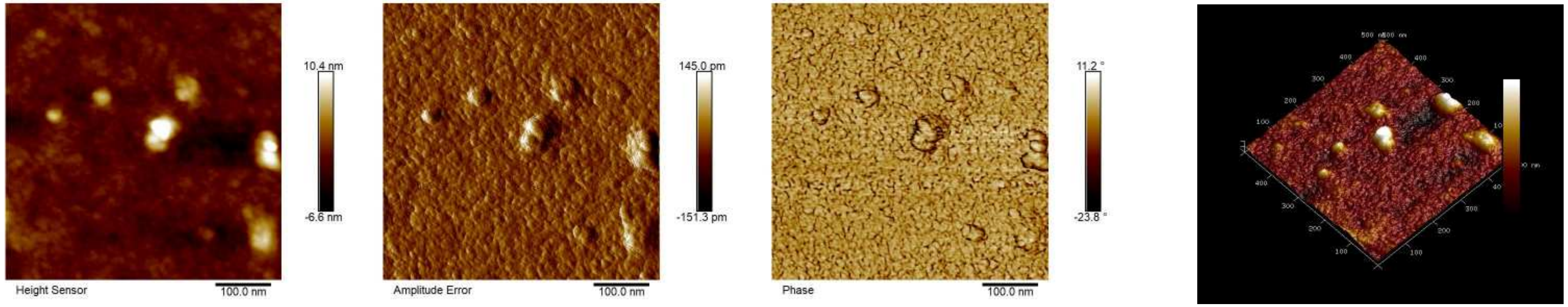

Area 3

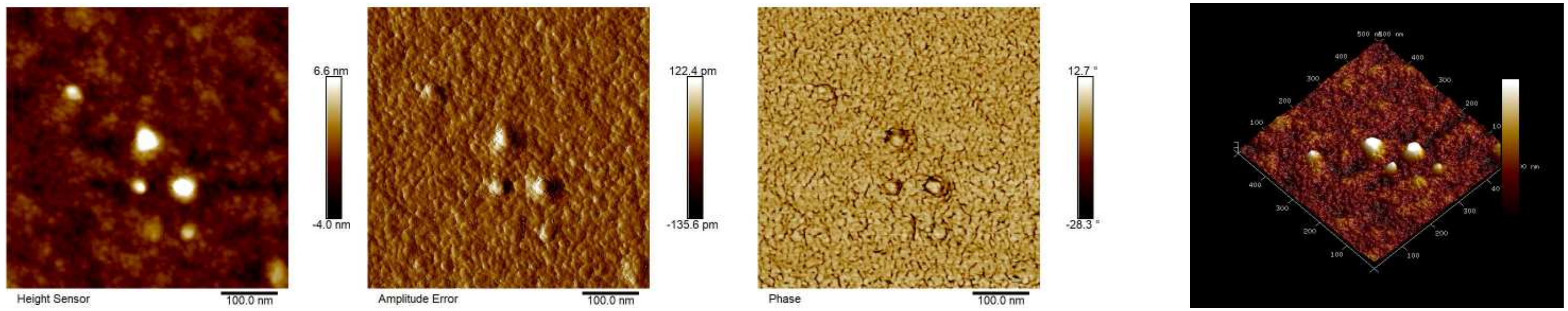

Teicoplanin Control 12.5 mg/ml – H_2O

Area 1

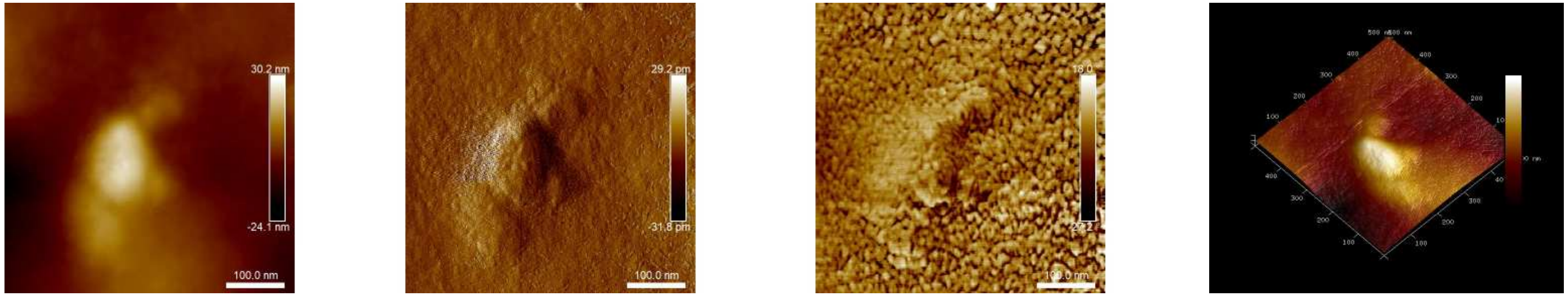

Area 2

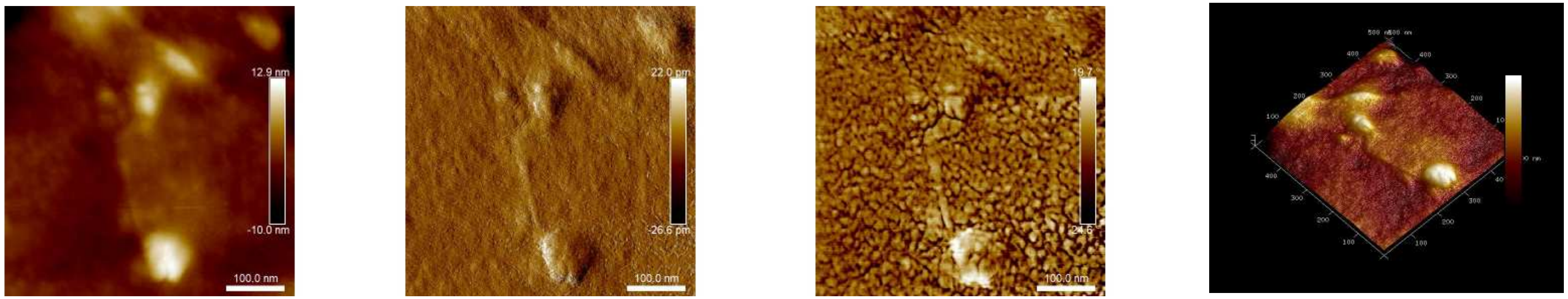

Area 3

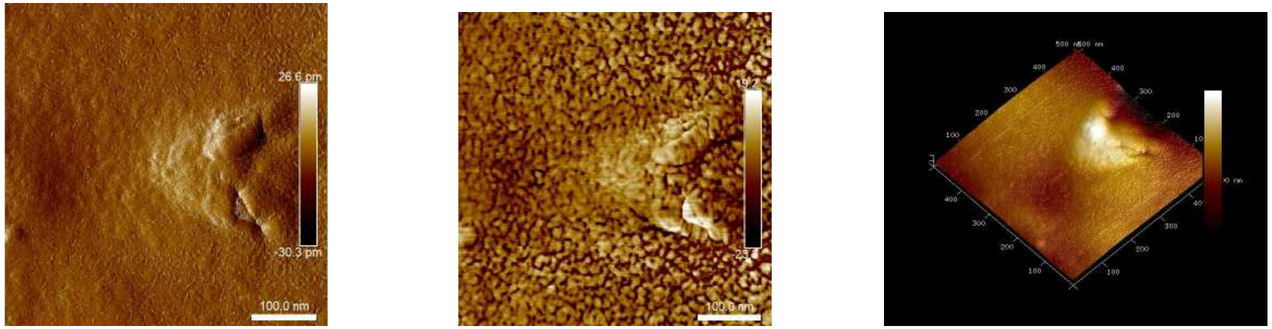

BSM Control 1 mg/ml – H_2O

Area 1

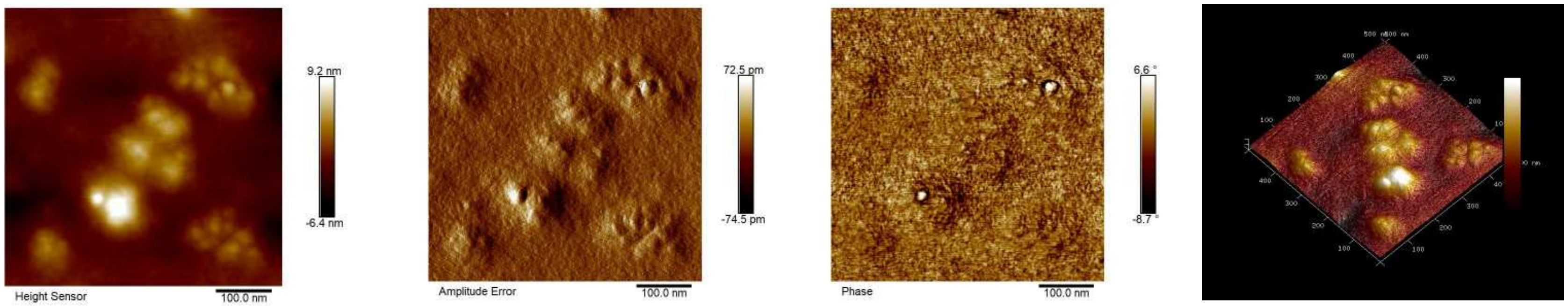

Area 2

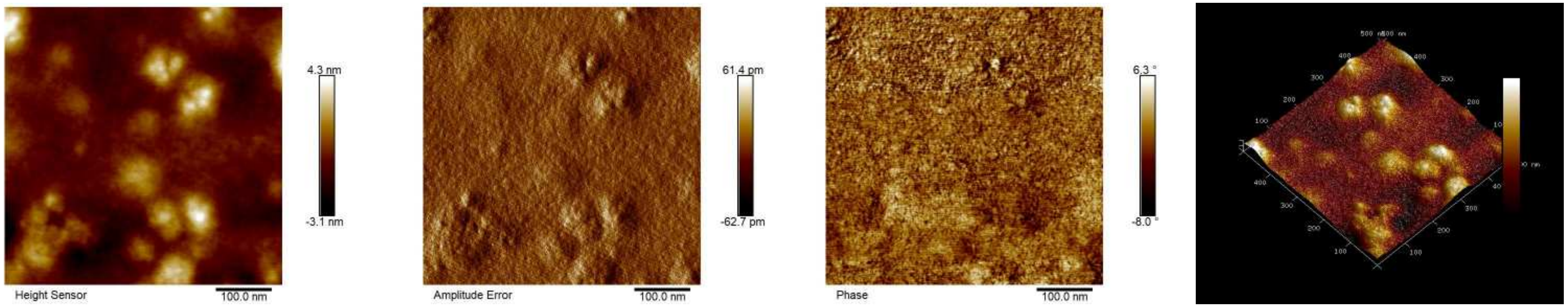

Area 3

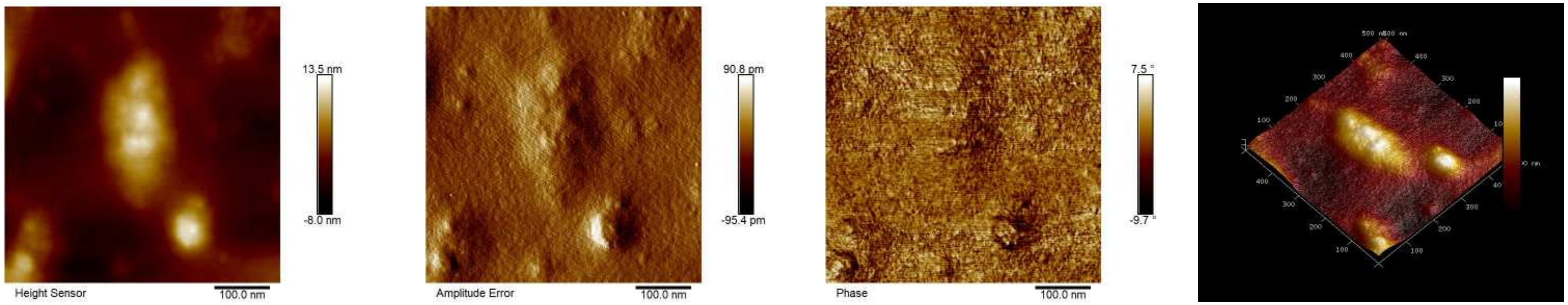

TP (0.125 mg/ml) - BSM (1 mg/ml) – H_2O

Area 1

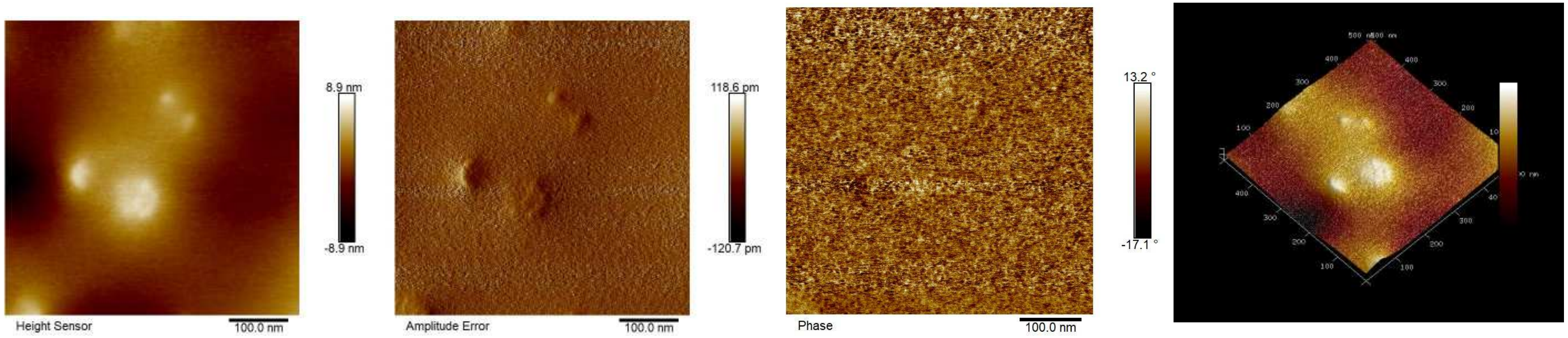

Area 2

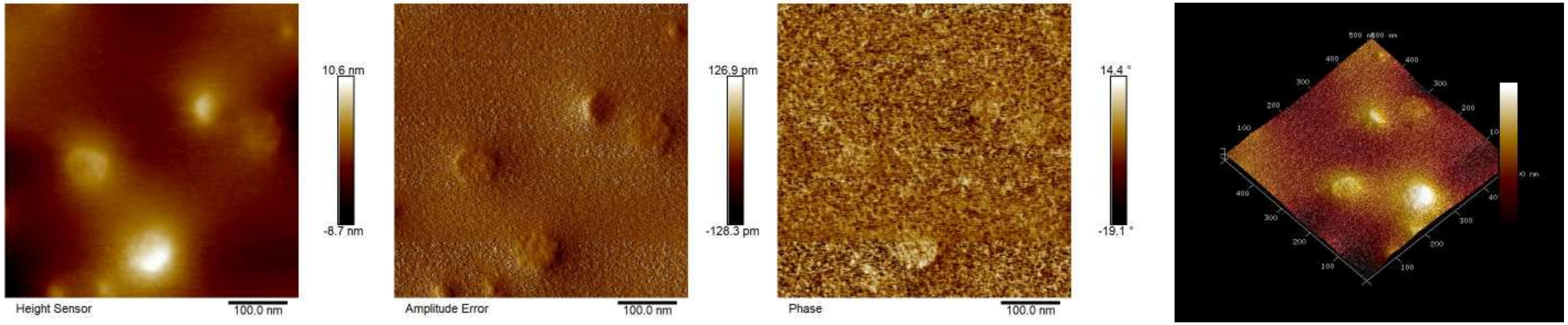

Area 3

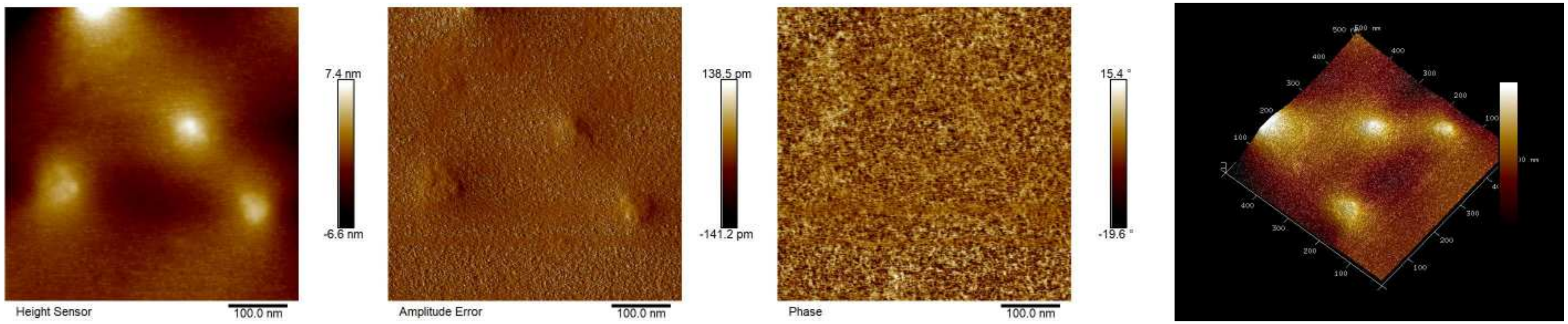

TP (1.25 mg/ml) - BSM (1 mg/ml) – H_2O

Area 1

Area 2

Area 3

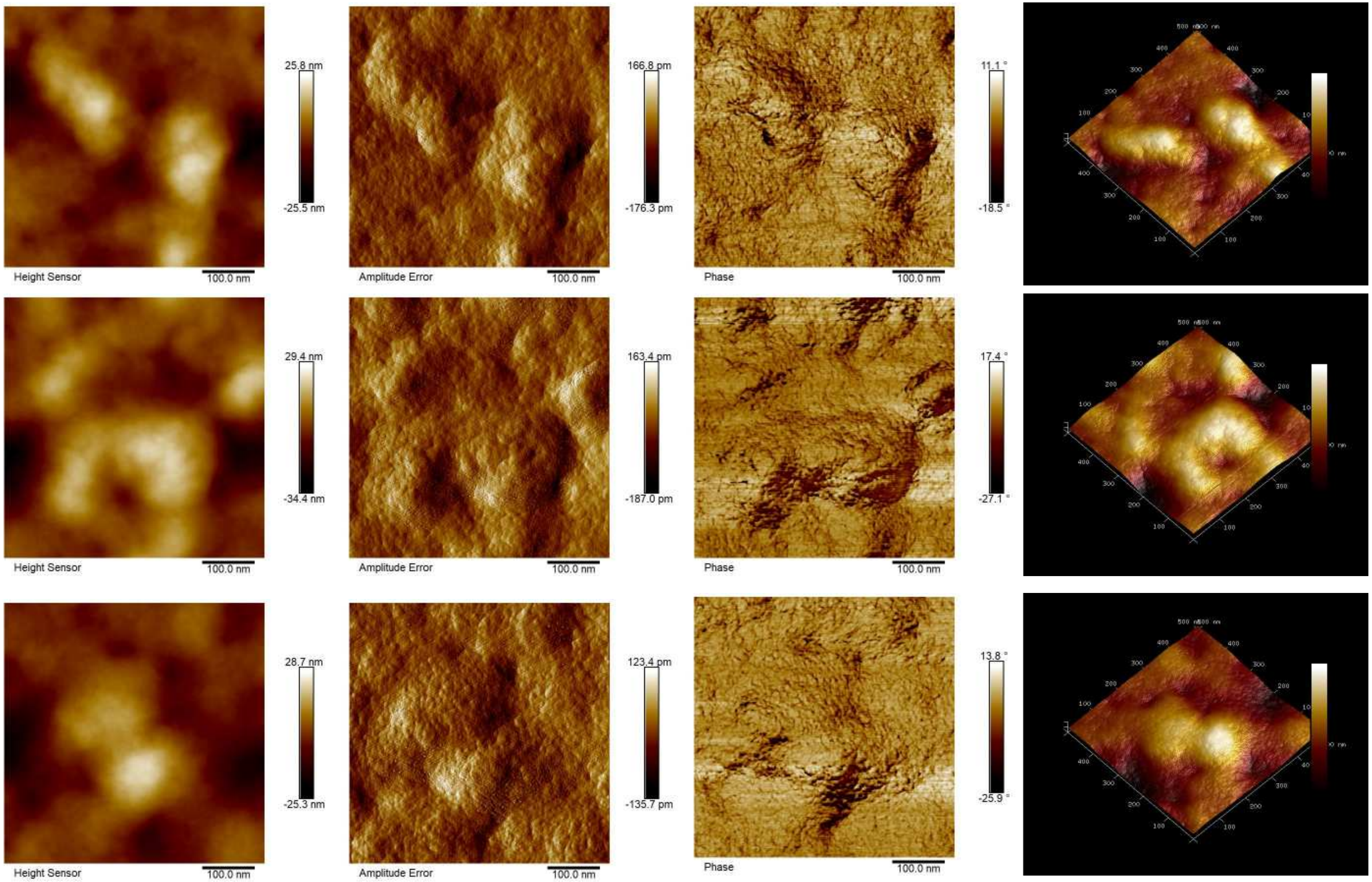

TP (12.5 mg/ml) - BSM (1 mg/ml) – H_2O

Area 1

Area 2

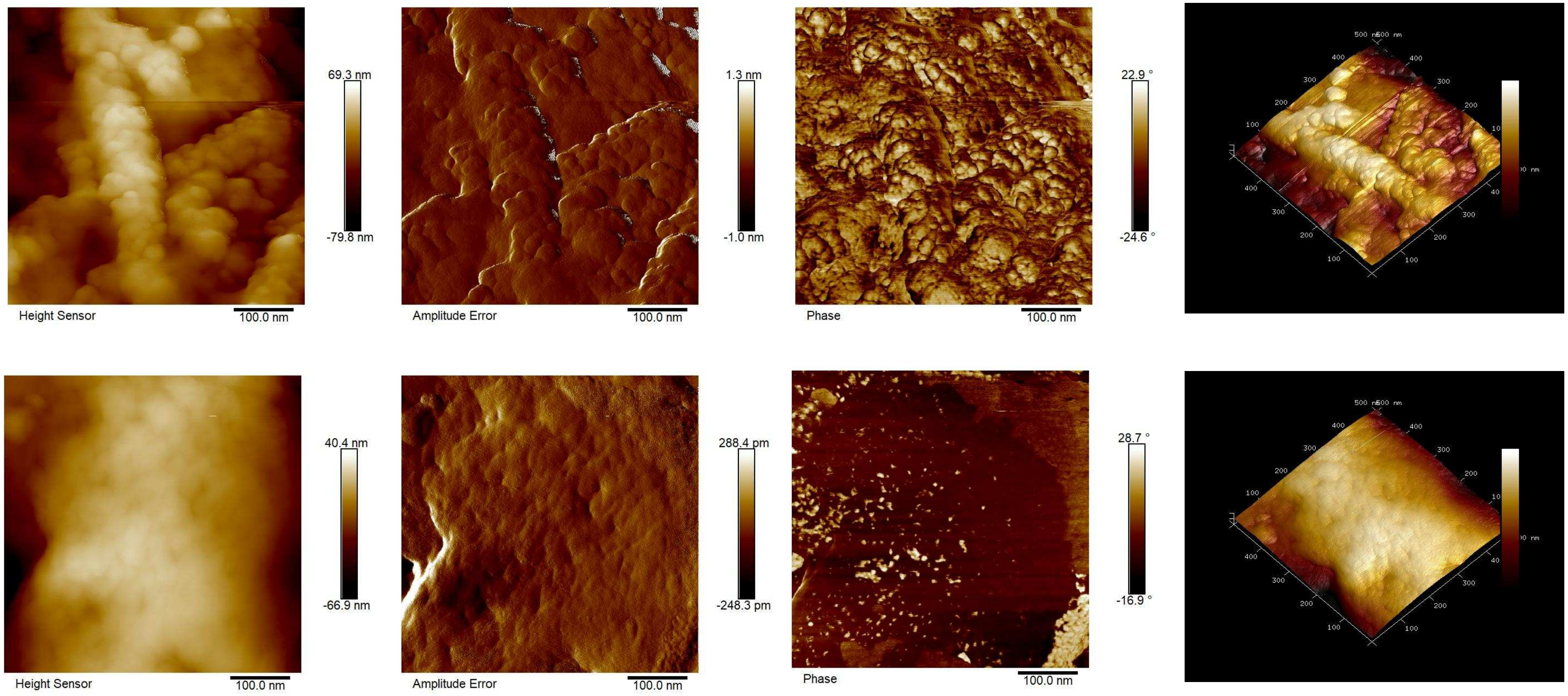

Additional TP (12.5 mg/ml) - BSM (1 mg/ml) – H_2O

Area 1

Area 2

Area 3

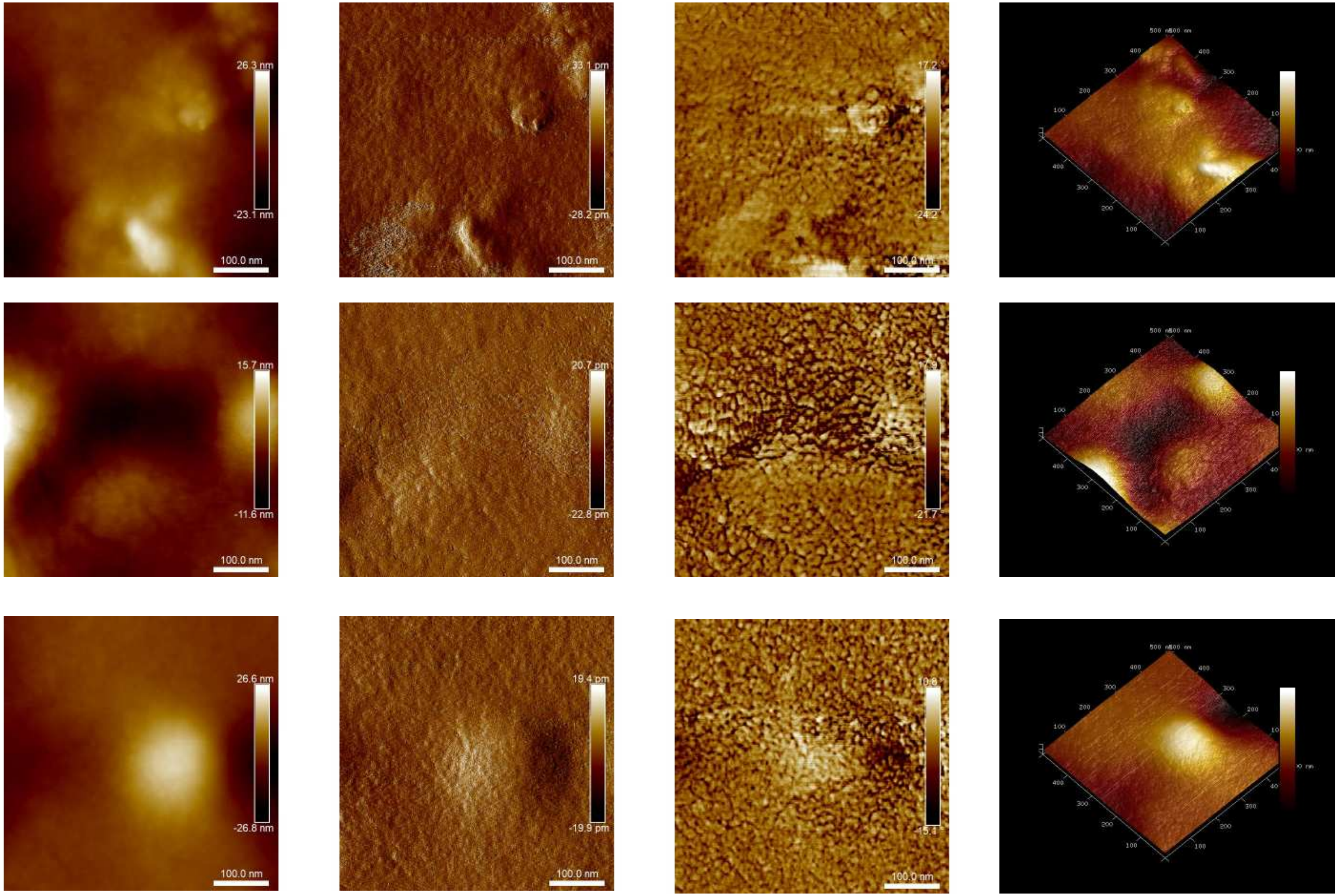

Supplement: Supplementary file 2 — Supplementary Information 2. [file 41598_2023_38036_MOESM2_ESM.pdf]
